# Epidemiological data of national Kawasaki disease registry in Iran, 2007–2019

**DOI:** 10.3389/fped.2022.988371

**Published:** 2023-01-11

**Authors:** Leila Shahbaznejad, Ali Hosseininasab, Leila Mahboobi, Hamid Mohammadi, Hassan Esmaeili, Seyed Majid Farrokhi Far, Mohammad Reza Alipour, Iraj Sedighi, Roxana Mansour Ghanaei, Behnam Sobouti, Alireza Taremiha, Abdol Reza Malek, Keyghobad Ghadiri, Gholamreza Soleimani, Hamed Tabasizadeh, Abdolkarim Ghadimi Moghadam, Manouchehr Barak, Forood Salehi Abarghouei, Houman Hashemian, Hossein Daryani Nezhad, Hamid Reza Sherkatolabbasieh, Masoumeh Abedini-Varamini, Mohammad Bagher Rahmati, Faezeh Sadat Movahedi, Mohammad Sadegh Rezai

**Affiliations:** ^1^ Pediatric Infectious Diseases Research Center, Communicable Diseases Institute, Mazandaran University of Medical Sciences, Sari, Iran; ^2^ Research Center of Tropical and Infectious Diseases, Afzalipour Hospital, Kerman University of Medical Sciences, Kerman, Iran; ^3^ Department of Pediatrics, School of Medicine, Ardabil University of Medical Sciences, Ardabil, Iran; ^4^ Neonatal Research Center, Namazi Teaching Hospital, Shiraz University of Medical Sciences, Shiraz, Iran; ^5^ Ischemic Disorders Research Center, Golestan University of Medical Sciences, Gorgan, Iran; ^6^ School of Medicine, Heshmatie Hospital, Sabzevar University of Medical Sciences, Sabzevar, Iran; ^7^ Cardiovascular Research Center, Afshar Heart Center, Shahid Sadoughi University of Medical Sciences, Yazd, Iran; ^8^ Department of Pediatrics, School of Medicine, Besat Hospital, Hamadan University of Medical Sciences, Hamadan, Iran; ^9^ Pediatric Infections Research Center, Research Institute for Children Health, Mofid Children's Hospital, Shahid Beheshti University of Medical Sciences, Tehran, Iran; ^10^ Department of Pediatrics, School of Medicine, Shahid Motahari Burns Hospital, Iran University of Medical Sciences, Tehran, Iran; ^11^ Department of Pediatrics, School of Medicine, Qods Teaching Hospital, Qazvin University of Medical Sciences, Qazvin, Iran; ^12^ Department of Pediatrics, School of Medicine, Akbar Hospital, Mashhad University of Medical Sciences, Mashhad, Iran; ^13^ Infectious Diseases Research Center, Research Institute for Health, Imam Reza Hospital, Kermanshah University of Medical Sciences, Kermanshah, Iran; ^14^ Children and Adolescents Health Research Center, Research Institute of Cellular and Molecular Sciences in Infectious Diseases, Zahedan University of Medical Sciences, Zahedan, Iran; ^15^ Department of Clinical Sciences, School of Medicine, Imam Hossein Hospital, Bahar Hospital, Shahroud University of Medical Sciences, Shahroud, Iran; ^16^ Cellular and Molecular Research Center, Yasuj University of Medical Sciences, Yasuj, Iran; ^17^ Cardiovascular Diseases Research Center, Birjand University of Medical Sciences, Birjand, Iran; ^18^ Department of Pediatrics, School of Medicine, 17 Shahrivar Hospital, Guilan University of Medical Sciences, Rasht, Iran; ^19^ School of Medicine, Islamic Azad University of Tonekabon, Tonekabon, Iran; ^20^ Department of Pediatrics, School of Medicine Shahid Rahimi Hospital, Shahid Madani Hospital, Lorestan University of Medical Sciences, Lorestan, Iran; ^21^ Department of Pediatrics, School of Medicine, Besat Hospital, Kurdistan University of Medical Sciences, Sanandaj, Iran; ^22^ Department of Pediatric Infectious Diseases, Children's Clinical Research Development Center, Hormozgan University of Medical Sciences, Bandar abbas, Iran

**Keywords:** kawasaki disease, registry, national, Iran, strawberry tongue

## Abstract

**Introduction:**

Kawasaki disease(KD) is a vasculitis of childhood that tends to influence the coronary arteries. There is no national data about the prevalence of KD in Iran. This study aimed to perform a national registry in Iran for 13 years.

**Methods:**

In this retrospective study, the data for KD extracted from medical records of <19 year-old patients admitted to tertiary hospitals in Iran between 2007 and 2019 were recorded in the national KD registry system. Age, admission date, gender, location, and presence of KD criteria, laboratory and echocardiography findings, and treatment modalities were evaluated. Complete KD was considered if ≥4 clinical criteria of the KD existed and otherwise, incomplete KD was considered.

**Results:**

Data from 1,682 KD patients including 999(59.39%) boys and 683(40.61%) girls and male/female ratio of 1.46 were evaluated. The mean age was 3.08 ± 2.49 years and 1465(87%) were living in urban regions. The yearly incidence of the disease was between 2.62 to 3.03 from 2015 to 2019. The highest age-specific incidence was observed in children <1-year-old. Incomplete and resistant KD included 1,321(78.54%) and 9(0.54%) patients, respectively. Abnormal echocardiography was detected in 619(36.80%) patients. Leukocytosis, with dominancy of neutrophils, anemia, thrombocytosis and increased ESR and CRP were the most noticeable laboratory findings. No death due to KD disease was reported.

**Conclusion:**

Based on this study, most of the KD cases are presented with atypical presentation in Iran. So, increasing awareness of primary healthcare workers by educating and updating their data is very important in timely diagnosis and management of the disease.

## Introduction

Kawasaki disease (KD) is a vasculitis of childhood that tends to influence blood vessels to become inflamed or swollen throughout the body in addition to coronary arteries (1, 2). The clinical manifestations of the disease include fever, mucocutaneous presentation, and lymphadenopathy. In the acute phase, the main clinical presentations include fever, conjunctivitis, rash, oral mucosal changes, cervical lymphadenopathy, and extremity erythema (3). The etiology of KD is unknown, but it is believed that an infectious agent and a genetic predisposition are involved (4). Measles, non-exudative pharyngitis, retropharyngeal abscess, bacterial cervical adenitis and scarlet fever are misdiagnosed with KD due to their similar manifestations (5).

About 25% of the patient may experience coronary artery lesions if remain untreated, especially during the acute phase. Myocardial ischemia and infarction due to thrombosis formation or rupture of aneurysms may occur; So, the sequel of the disease may last for a long time and influence the lifelong cardiovascular survival of the patients (3). High-dose intravenous immunoglobulin and salicylic acid (Aspirin) are approved treatments for the disease (2). Other alternatives or adjuncts include corticosteroid, infliximab, etanercept, plasmapheresis in addition to a secondary IVIG infusion among KD resistant patients (6). Appropriate management improves cardiac outcomes and may prevent coronary abnormalities (2, 7).

Since 1967, when the first report of KD was published in Japan, there have been other reports worldwide (8, 9). The KD incidence is different throughout the world with the highest rate in Japan, Korea, and Taiwan (8–12) and it is more prevalent in Asian background populations (3, 9). The main etiology of the KD is unknown, but due to the ethnic and geographical tendency of the disease, some environmental factors or the genetic basis of the illness may be considered (3, 9). In addition to the geographical distribution of the disease, the seasonal pattern of KD in some reports raises the probability of an infectious agent, which may be more prevalent in particular seasons or months (13).

Japan reports the incidence of KD biannually. Also, there are scattered reports of the national or regional incidence in the rest of the world (8–10). In Iran, the exact prevalence of the disease is unknown because most of the reports are sparse and local (14, 15). No national data in Iran exists about the prevalence of the KD; So, we performed this national registry for KD patients as the first registry reporting the epidemiological characteristics of the KD patients over 13 years.

## Methods

### Study design

In this retrospective study, the data of the national KD registration system which were extracted from hospital records were evaluated to determine the incidence of Kawasaki disease in Iran. This registration system has been designed to record data from KD and acute rheumatic fever patients in Iran in 2018. Data for the minimum dataset (MDS) in the registry program about KD had been obtained from a combination of literature review, data collection from patients' medical records, and an expert panel approach (16).

### Data collection

The Iranian Kawasaki Disease registry was created at March 2018 in Mazandaran University of Medical Sciences to determine the prevalence, patient demographics including age at diagnosis, admission date, gender, location, and presence of clinical diagnostic criteria of KD (3) including non-purulent bilateral conjunctivitis, cervical lymphadenopathy, rash, extremity changes (erythema, induration or pilling) and oral mucosal changes (strawberry tongue, cracked red lips or oral mucosal erythema), laboratory and echocardiographic data and treatment modalities. If ≥4 clinical criteria of the KD were recorded in the system, the patient's final diagnosis was complete KD; in the cases with less than four criteria, incomplete KD was considered (2). The patients who received more than one standard dose of IVIG due to persistence of fever 36 h after the end of IVIG infusion were considered to be treatment resistant (17).

The study enrolled hospital records of patients less than 19 years of age with final diagnosis of Kawasaki disease (International Classification of Diseases (ICD)-10 coding for KD: M30.3, Mucocutaneous lymph node syndrome [Kawasaki]) admitted to 29 tertiary educational hospitals in Iran. Retrospective medical data retrieval from 13 years (March 2007–2019) was collected and reviewed. A research electronic database capture was created and sent to the participant clinicians together with the study protocol.

### Ethical considerations

The ethics committee of Mazandaran University of Medical Sciences approved the study protocol (#IR.MAZUMS. REC.1397.2759). All data was published in general and anonymous.

### Statistical analyses

Data analysis was performed by SPSS software, version 20.0. Qualitative data were shown with frequency and percentage, and mean ± standard deviation was used for qualitative data. *P* values <0.05 were considered being statistically significant. The incidence of the disease was calculated per 100.000 population younger than 19 years in the region, which covered each medical center. Age-specific incidence was also calculated based on the frequency of population in age groups younger than one-year, 1 to 5 years, and >5 years per 100.000 population in each age group.

## Results

Nationwide, 1,682 KD cases including 999 (59.39%) boys and 683 (40.61%) girls and male/female ratio of 1.46 were included in the registry. The mean age of the patients was 3.08 ± 2.49 years with a median [IQR] of 2.25 [1.25–4.25], and a mode of 1 year. The youngest child was a 31 days old boy and the oldest one was a 17 years old girl, both of them presented with the complete manifestation of KD. Among children with a diagnosis of KD, 1465 (87%) were living in urban regions. 309 children (18.98%) were older than 5 years, 1,028 (63.14%) were between 1 and 5 years and 291 (17.87%) were less than one year old. Yearly age-specific incidence was calculated in 100.000 populations in each age group and ranged between 7.28 in the children <1 year to 0.78 in those >5 years of age (Table 1).

**Table 1:** The age-specific incidence of the Kawasaki disease in the last 5 years of the study (2015–2019).

Year	The incidence rate in children <18 years (×100.000)
2015	2.62
2016	2.99
2017	2.78
2018	2.81
2019	3.03
Age group	Mean 5-year incidence of KD in each age group (×100.000)
<1 year	7.28
1–5 year	5.07
>5 year	0.78

**Figure 1:**
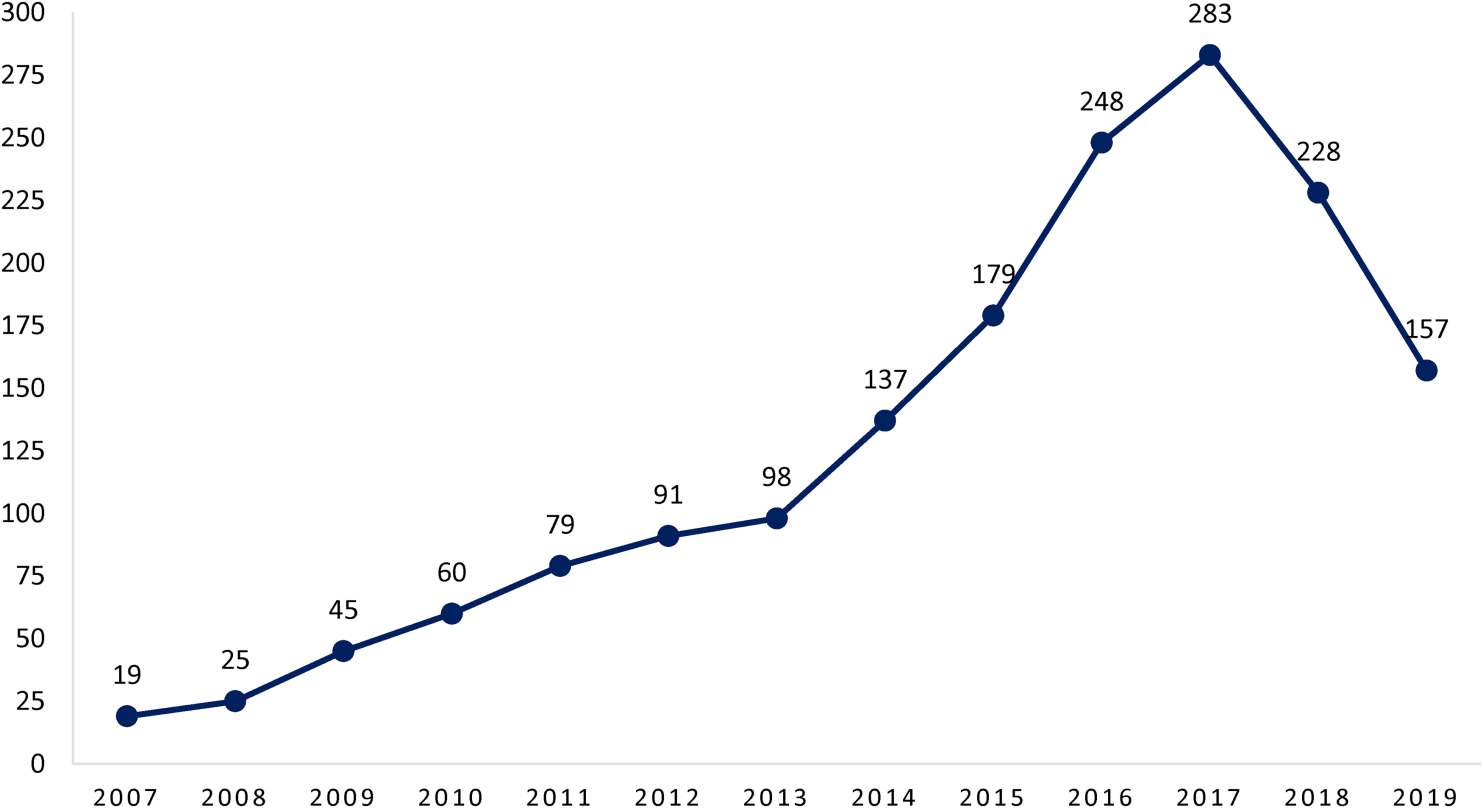
Yearly incidence of the KD in 100,000 populations <18 years from 2007 to 2019.

The number of patients increased gradually from the first year of the study (Figure 1) and reached its highest value in 2017 (Figure 1). The incidence of the disease was calculated based on the number of patients in the last 5 years of the study when the incidence of the disease became steadily state. The yearly incidence rate of the disease in 100.000 populations <18 years was between 2.62 to 3.03 from 2015 to 2019 (Table 1).

**Figure 2:**
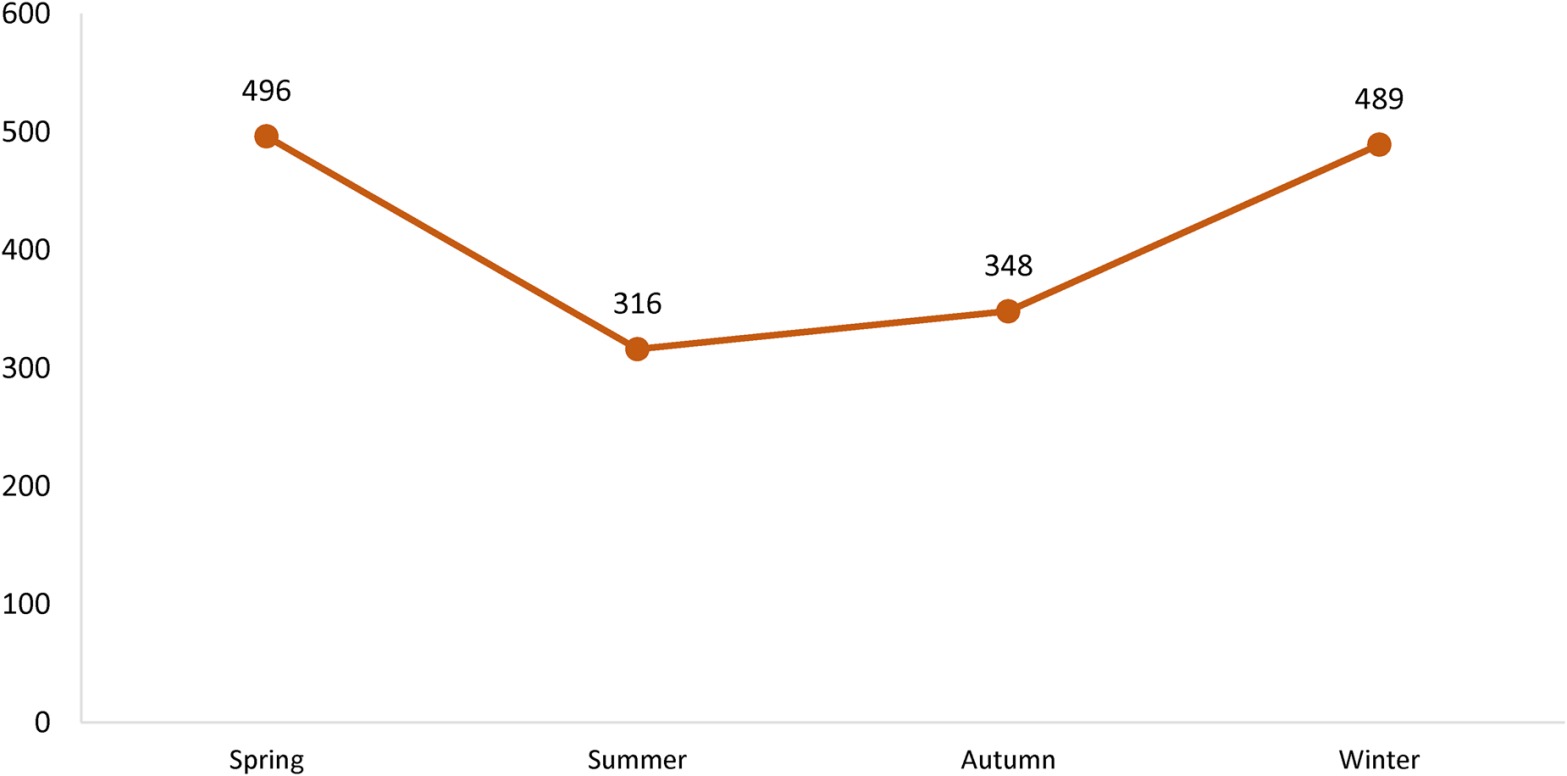
Seasonal distribution of the Kawasaki disease in the country between 2007 and 2019.

**Figure 3:**
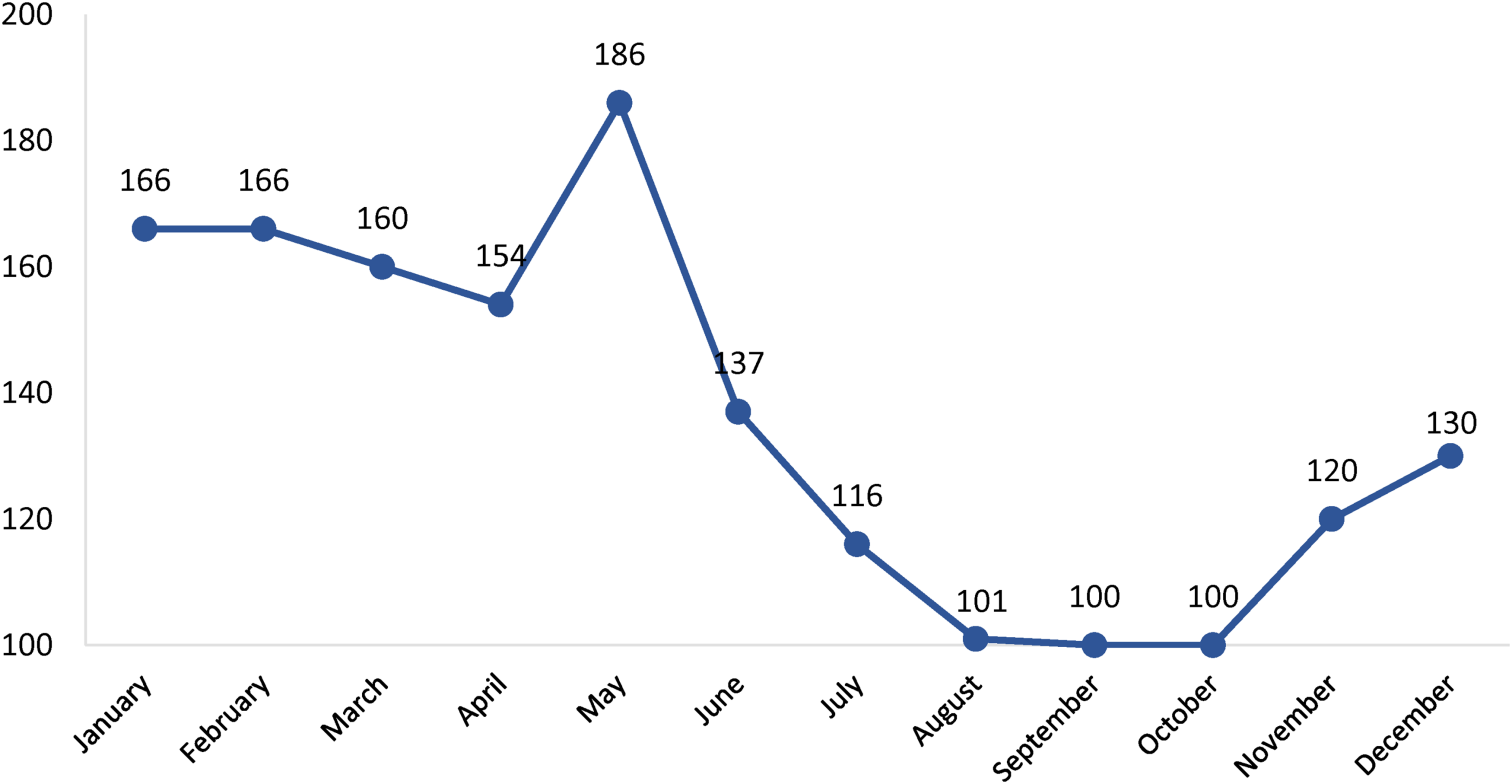
Monthly distribution of the Kawasaki disease in the country between 2007 and 2019.

The seasonal and monthly incidence of the disease is noted in Figures 2, 3. The mean age-specific incidence of the KD in each age group was also calculated for the last 5 years (Table 1). The highest age-specific incidence was observed in children younger than one-year.

A total of 361 cases were classified as complete (21.46%) and 1,321 as incomplete (78.54%) KD. Echocardiographic examinations were performed during the acute phase of KD in most of the patients but it was available for 1,235 patients (73.4%), among which 619(36.80%) were found to have an abnormal echocardiogram including pericardial, myocardial, valvular, and coronary artery abnormalities. Coronary artery abnormalities were the most frequent findings in 390 (63%). Of these, dilatation was the most common lesion, diagnosed in 182 patients (29.4%). Coronary aneurysms were diagnosed overall in 120 patients (19.39%). Table 2 shows the frequency of abnormality in echocardiography in detail.

**Table 2:** Echocardiography abnormalities in KD patients suffering from cardiac involvement (N: 619).

Type of abnormality	Frequency (%)
Valve involvement	158 (25.53)
Myocarditis	9 (1.45)
Pericarditis	75 (12.12)
Coronary artery abnormalities	390 (63.00)
Aneurysm	120 (19.39)
Dilatation	182 (29.40)
Ectasia	72 (11.63)
Shining	60 (9.69)
Lack of tapering	13 (2.10)
Other	91 (14.70)

Nine patients (0.54%) needed more than one standard dose of IVIG and were considered resistant KD which glucocorticoids (Methyl prednisolone) was prescribed for 2 of them (22.22%).

On laboratory evaluation, leukocytosis, with dominancy of neutrophils, anemia, and thrombocytosis in addition to increased erythrocyte sedimentation rate and C-reactive protein were the most noticeable findings. Hyper-transaminasemia, hypoalbuminemia, and pyuria were common. Laboratory characteristics of the patients are shown in Table 3. Pyuria with ≥5 WBC in the urine sample was reported in 228 (13.56%) patients while 50 (2.97%) of them showed microscopic hematuria (≥5 RBCs in the urine sample). No death due to KD disease was reported.

**Table 3:** Laboratory characteristics of the patients with the diagnosis of KD (*N *= 1682).

Laboratory test	Mean ± SD	Median [IQR]	Mode	Min, Max		N (%)
CBC						
WBC	13.22 ± 6.05	12.20 [9.10–16]	12	1.50, 71.80	WBC < 5,000	41 (2.44)
					WBC > 15,000	431 (25.62)
Nuet	8.20 ± 4.78	7.63 [4.88–10.80]	8.40	0.61, 34.96	Neut > 10,000	220 (13.08)
Lymph	4.25 ± 2.49	3.70 [2.43–5.56]	2.73	0.19, 18.67	Lymph < 1,000	17 (1.01)
Mono	0.57 ± 0.60	0.40 [0.24–0.71]	0.23	0.03, 6.67		
Eo	0.37 ± 0.33	0.27 [0.17–0.46]	0.18	0.02, 3.59		
Hb	10.42 ± 1.48	10.40 [9.50–11.30]	11	5.7, 19.1	Hb < 10	524 (31.15)
PLT	459,864.60 ± 209,002.33	435,000 [314,000–574,000]	456,000	24,000, 1,927,000	PLT > 450,000	635 (37.75)
					PLT < 100,000	18 (1.07)
ESR	75.42 ± 31.19	76.50 [53–99]	70	1, 161	ESR > 30	1,273 (75.68)
CRP	35.97 ± 37.37	20 [10–40]	10	0.3, 293.4	CRP > 10	816 (48.51)
Na	136.83 ± 3.65	137 [134–139]	138	122, 156	Na < 130	6 (0.36)
Alb	3.88 ± 0.56	3.9 [2–5.60]	3.80	2, 5.60	Alb < 3	34 (2.02)
SGOT	55.32 ± 218.42	33 [24–49]	25	7, 5560	SGOT > 35	481 (28.60)
SGPT	50.32 ± 126.43	25 [15–47]	10	2, 3430	SGPT > 35	350 (20.81)

## Discussion

This study is the first national registry about Kawasaki disease in Asia except Japan and South Korea (8, 10) and the first and unique report in the Middle East region. The study duration is considerable and consists of 13 consecutive years. Kawasaki disease was first reported in Japan and nowadays, most of the cases are from Japan and the far eastern region (8). KD is relatively rare with different incidences between ethnic groups and higher rates in children of Asian ethnicity (3, 4, 18).

In the first years of the study, the incidence of KD was very low, but during the time, the number of febrile children with KD diagnosis increased due to proper training of physicians and improved awareness of the disease to discern patients at risk and promptly and adequately refer them for further diagnostic workup and treatment and also availability of diagnostic tools all over the country (19). On the other hand, most of the KD cases were atypic and the diagnosis was based on factors like the presence of a pediatric cardiologist, and echocardiography instruments (3). Both factors may play a role in many countries, particularly in those countries where KD has been described more recently (20). In our registry, in the first year (2007), only 19 cases were recorded, but after that, the number of cases increased dramatically. The yearly incidence of the disease is increased over the years from 2.63 cases per 100.000 population <18 years in 2015 to 3.03 in 2019.

In this study, the incidence of KD in <5 years old children was 27.16 in 100.000 from 2015 to 2019. The annual incidence of KD in Japan in 2015 was 330/100.000 children <5 years and 309 in 2016 (8). This annual incidence was 113 in 100.000 children <5 years in Korea from 2006 to 2008 and 69 in Taiwan from 2003 to 2006 (10, 11). The annual incidence of KD in 100.000 children <5 years of old in the United States was 20.8 in 2006 (18) and Canada 20.6 between 1998 and 2000 (9). Although, the lower incidence of our study may be due to referring patients to other medical centers and not registering their data in our system.

In our study, most KD cases were <5 years old, and the incidence of the disease was considerable in children younger than 1 year. Similar to us, KD generally occurs in children <5 years old (11) and in other studies, especially those from Japan and Korea, most cases were <5 years (8, 10). There is no data on the incidence of KD from neighboring countries. In comparison with other reports, nearly two-thirds of our patients were boys and the boy/girl ratio was 1.46. This ratio was 1.34 in Japan (8) and 1.47 in Korea (10). Similar results were reported from another region (18). KD occurs more commonly in the male gender (11). Susceptibility of boys to some environmental factors or infections can justify this gender dominance.

In our study, cases were more admitted in winter and spring and the highest monthly admission was between January and May. In Korea, most of the cases were from winter and summer (10). However, in the USA, the frequency of admitted cases from December to March was higher than in other months (18). The peak months of KD in Taiwan were during the summer months of April, May, and June (11). Seasonal wind is supposed to bring the probable KD agent across the Northern Hemisphere (21).

In this study, only one-fourth of the patients fulfilled the complete KD criteria, and most of them showed incomplete presentations of the disease. Researches from Japan and other regions of the world (8) illustrate the dominancy of typical cases, as in Japan 77.8% of cases were typical. Some of our incomplete diagnosis may be due to a lack of data during hospitalization, but the ratio of incomplete cases is considerable as we have experienced in the country.

We found a considerable number of patients with positive echocardiographic findings, and coronary artery abnormalities were the most common reports in this study so that 29.4% of the patients had dilatation and coronary aneurysms were found in 19.39%. In the study by Gorrab et al. in Maghreb countries, KD in Maghrebi children residing in Quebec were compared with Tunisia, Morocco, and Algeria. Coronary artery dilation and aneurysms affected 39% of the patients in Quebec, 22% in Morocco, 19% in Tunisia, and 26% in Algeria (22). The incidence of coronary artery aneurysm was 8%–12% in KD groups receiving either IVIG and/or Aspirin or none of them (11). Also, coronary aneurysms were reported 8.5% between 2000 and 2011 in Portugal (23). The most important sequel of the KD is cardiovascular damage (3). Since most of our patients were incomplete KD and the diagnosis was mostly based on echocardiographic abnormalities, this high proportion of coronary artery abnormalities is predictable.

Laboratory abnormalities were common in KD patients; depending on the time of the disease, an increase in ESR and CRP, leukocytosis with neutrophilia is predictable in these patients, although some patients may show different results. Involvement of hepatobiliary and urogenital systems was also seen in KD.

We had some limitations in our study. Since KD is a vasculitis with a self-limiting acute phase in which a fever and clinical symptoms subsided even without specific treatment, some cases may have been misdiagnosed. In this report, we tried to report the true incidence of KD in Iran but this number is not the exact number in the country, because data from some hospitals were not accessible to the investigators. In addition, some patients may not have been referred to the hospitals in acute phase of the disease and were not admitted. For this reason, we adjusted and reported the incidence of the disease based on the formal population number of people younger than 19 years in just logged regions. So, the true incidence of the disease is much higher than this report. Due to the COVID-19 pandemic, we limited our study to the end of 2019. So, we were unable to find the trend of the disease in the following years.

## Conclusion

Based on this study, most of the KD cases are presented with atypical presentation in Iran. So, increasing awareness of primary healthcare workers by educating and updating their data is very important in timely diagnosis and management of the disease.

## Data Availability

The original contributions presented in the study are included in the article/Supplementary Material, further inquiries can be directed to the corresponding author/s.
